# Plastic Behavior of Metallic Damping Materials under Cyclical Shear Loading

**DOI:** 10.3390/ma9060496

**Published:** 2016-06-21

**Authors:** Chaofeng Zhang, Longfei Wang, Meiping Wu, Junhua Zhao

**Affiliations:** Jiangsu Key Laboratory of Advanced Food Manufacturing Equipment & Technology, Mechanical Engineering School of Jiangnan University, Wuxi 214122, China; zcf830703@163.com (C.Z.); wx333vip@126.com (L.W.); junhua.zhao@163.com (J.Z.)

**Keywords:** low yield strength steel, cyclic shear loading, failure mechanism, fatigue performance, stability, large plastic shear strain

## Abstract

Metallic shear panel dampers (SPDs) have been widely adopted in seismic engineering. In this study, axial and torsional specimens of four types of metallic damping materials, including three conventional metallic steels as well as low yield strength steel 160 (LYS160), were tested in order to investigate the material response under repeated large plastic strain and low cycle fatigue between 10 and 30 cycles. The present study demonstrated that both the deformation capacity and fatigue performance of LYS160 were underestimated by the conversion from the traditional uniaxial tensile test. The main difference in the failure mechanism between LYS160 and the three conventional materials was determined from the scanning electron microscopy data. The dominant failure mode in LYS160 is stable interlaminate slip and not bucking. Our results provide physical insights into the origin of the large deformation capacity, which is an important foundation for the lightweight design of SPDs.

## 1. Introduction

Metallic dampers, categorized as passive dampers, are widely adopted in seismic structural systems. The earthquake energy is mainly dissipated by cyclical axial tension-compression, bending, and shearing of the metallic dampers. When the metallic dampers are under tension and compression loading, they are prone to necking and instability, which leads to the poor deformation capacity of the damper. Restraint components were generally introduced to improve the stability of the braces [1]. In addition, the sufficient resist force and stiffness of the damper can also be provided by optimization design when the metallic damper is under bending [2]. Each damper has its own advantage. Here, the importance of shear panel dampers (SPDs) is emphasized as it has aroused attention of researchers in recent years [3,4].

The stability of the shear panel is an important factor in ensuring high performance of the SPD [5]. When the panel is thin or the width-to-thickness ratio of the panel is high, out-of-plane buckling is likely to be produced, which is not beneficial for achieving a high deformation capacity [6]. In general, two methods are used to improve the stability of the SPD. One method involves adoption of high-strength buckling restraining components [7,8]. The distance between the buckling restraining components and the energy dissipation panel is rarely small. Although out-of-plane shear buckling can be inhibited, the improvement in the deformation capacity of the SPD is limited. Another method involves reduction of the width-to-thickness ratio of the shear panel using transverse [9], vertical [10], or cross stiffeners [11,12]. However, the presence of welding seams, which accompanied the stiffeners, prevents the improvement of the deformation capacity of the SPD. Thus, it is better to improve the deformation capacity of moderate shear panels using a low width-to-thickness ratio [13]. 

The corner stress concentration is very high when the width-to-thickness ratio of the shear panel is low [14,15]. Design optimizations, such as strengthening the weak part of the panel and weakening the strong part of the panel, have been traditionally used to improve the strain distribution. Accordingly, profiled panels [16] and transition arcs [17] at the panel corners were designed. However, these two methods do not work when the plastic deformation of the SPD is very large. The deformation capacity of the SPD can be considerably improved when the corner stress concentration is suppressed by the ribs. In addition, since the welding seams located at the ends of the ribs are prone to cracking, stiffeners can be introduced to prevent overlapping of the plastic hinges and the ends of the ribs. The performance of the developed SPD was fairly stable even under constant dynamic loading [18] or random dynamic loading [19].

When an incremental shear strain loading sequence of ±5% is applied, the ultimate shear strain of the SPD is around 60%, which is approximately equal to the elongation of the SPD material. This implies that the ultimate deformation capacity, as well as the failure mechanism, cannot be evaluated by monotonic tension, cyclic tension-compression [20], or cyclic bending [21]. Lack of knowledge about the ultimate deformation capacity of the damping material makes it very difficult to judge the pros and cons of the SPD lightweight design. The loading condition of the SPD is approximately equal to the simple shear, which is the same as the loading condition of the torsional tests. Therefore, it is necessary and reasonable to investigate the failure mechanism and the ultimate deformation capacity of the metallic material by torsional tests. In order to take into account the actual working conditions of the damper, the present study considered the ultimate deformation capacities of the material when the low cycle fatigue numbers are between 10 and 30.

To meet the increasing need for improved damper performance, materials such as aluminum [22] and low yield strength steel (LYS) were adopted or developed for SPDs [23]. Although stainless steel 316 (SS316) is not currently used as a metallic material for SPD [24], it has been tested and analyzed in the present work since its deformation capacity is much larger than that of ordinary carbon steel (Q235). Hence, four types of metallic materials, namely aluminum 6061 (AL6061), low yield strength steel 160 (LYS160; yield strength = 160 MPa), Q235, and SS316 were researched and compared.

Traditional studies have shown that an SPD has advantages of compact structure and excellent performance through a reasonable design. However, a detailed investigation of the damping materials is important in order to optimize the SPD design. Therefore, in this study, axial and torsional specimens were tested to investigate the available ultimate shear plastic strain under low cycle fatigue. The failure mechanism was also analyzed by scanning electron microscopy (SEM). Our results provide physical insights into the origin of the large deformation capacity, which is important for the lightweight design of the damper.

## 2. Test Procedure

### 2.1. Test Setup and Specimen

**Figure 1:**
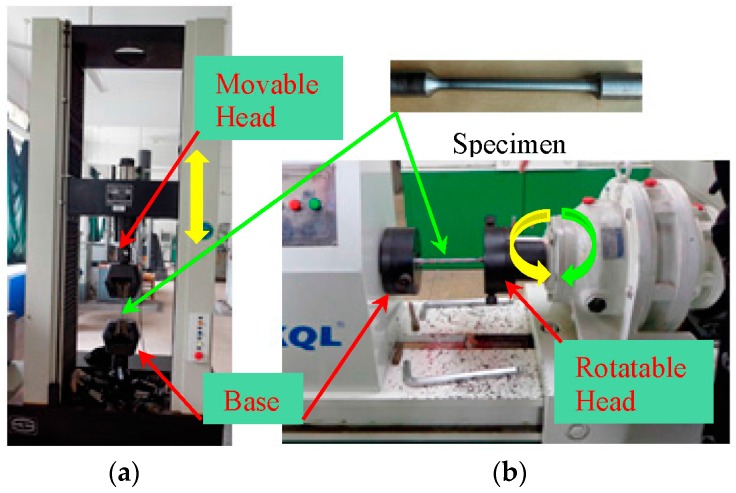
Test setup. (**a**) Tensile testing machine; (**b**) Torsion testing machine.

Figure 1 shows the experimental setup. The displacement control method was applied by a computer-controlled actuator (INSTRON, Boston, MA, USA; capacity = 250 kN) to the tensile specimens quasi-statically via a movable head (Figure 1a). One end of the torsion specimen was fixed to the base and the other end of the specimen was connected to the rotatable head of the torsion testing machine (Figure 1b). The monotonic tensile and torsion tests were conducted using these two machines, respectively. Cyclical torsion specimens were rotated forwards or backwards according to the negative and the positive rotation of the driving motor (Figure 1b).

An overview of the test specimen is also presented in Figure 1. The standard specimens were machined as round bars with a reduced cross-section. The reduced section diameter was not notched and maintained at 10 mm with a reduced length of 100 mm. Transition arcs between the reduced and unreduced sections were used to avoid stress concentration. The shapes and sizes of the specimens for all the monotonic and cyclical tests were identical [25].

### 2.2. Test Plan

#### 2.2.1. Monotonic Test

As listed in Table 1, 24 standard specimens of four different materials were designed to investigate their performance under monotonic tensile and shear loading. Clarification of the maximum performance of the metallic material is crucial for the lightweight design of the SPD. Therefore, the maximum shear strain and the maximum shear stress of the specimens were calculated from the angle-moment curve of the torsion testing machine using the following equation:

(1) $$\gamma =\frac{\theta \times \pi D}{360^{\circ }\times L}\times 100\%$$

(2) $$\tau_{\max}=\frac{3}{4}\frac{T}{W_{t}}$$

where *γ* is the shear strain, *θ* is the torsion angle, D is the diameter, *L* is the length, *T* is the torsion moment, and $W_{t}=\frac{\pi }{16}D^{3}$ is the torsion section modulus.

#### 2.2.2. Cyclical Shear Test

**Figure 2:**
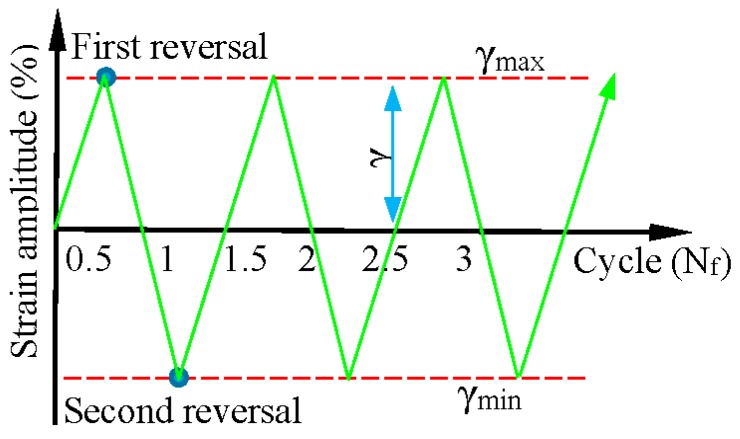
Loading pattern.

The load was applied by the shear strain (converted from the torsion angle) control, and the loading pattern is shown in Figure 2. The cycles were fully reversed with strain ratio R = “*γ*_min_/*γ*_max_” = −1, and only the strain amplitude was varied. According to the different seismic design codes, the maximum response cycles of the metallic damper range from 10 to 30 cycles. Along with the monotonic test results, the loading shear amplitudes were obtained as long as the fatigue cycles were not more than 30. The applied shear strain amplitudes of each material are listed in Table 2. One fatigue cycle represents a complete reciprocating motion. Thus, the corresponding fatigue cycle numbers at the first reversal and the second reversal are 1/4 and 3/4 fatigue life, respectively.

## 3. Results and Discussion

### 3.1. Monotonic Test

**Figure 3:**
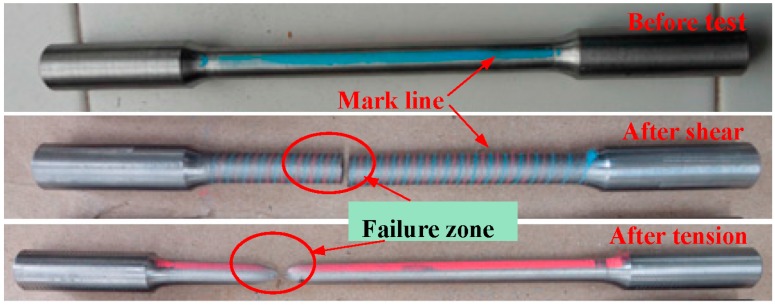
Images of the specimens before and after the tests.

The specimens showed different failure modes for tension and torsion tests (Figure 3). In order to demonstrate the failure details of the specimens more clearly, the overall failure was replaced by the local failure or the fracture face. Some important features of the fracture surface of the specimens were also observed by SEM. The mark lines were also labeled for observing the test results conveniently.

#### 3.1.1. Failure Mode

**Figure 4:**
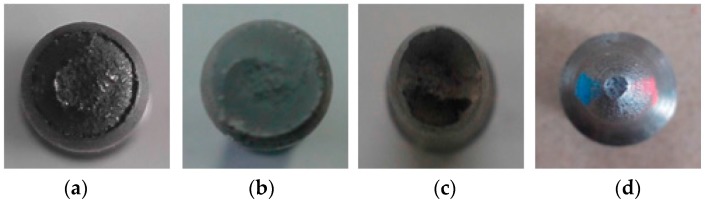
Failure modes under tension. (**a**) Q235; (**b**) AL6061; (**c**) SS316; (**d**) LYS160.

**Figure 5:**
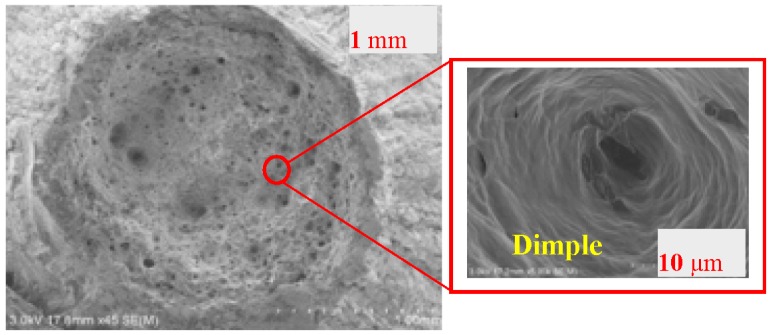
Fracture section of LYS160.

It is well known that the failure stages of a material under monotonic tension include elastic deformation, plastic deformation, necking, and terminating fracture. As shown in Figure 4, the fracture section of LYS160 was the smallest, and represented the largest section shrinkage. As the elongation of the specimen is positively correlated with the section shrinkage in a significant way, it can be understood that LYS160 possesses the maximum elongation among the four materials. In addition, the fracture morphology of LYS160 was a dimple pattern with features of plastic fracture, which was also different from the rest of the specimens (see Figure 5). A large number of the dimples were distributed on the fracture face. The dimples were large and deep. Both of these phenomena implied sufficient plastic deformation of LYS160.

**Figure 6:**
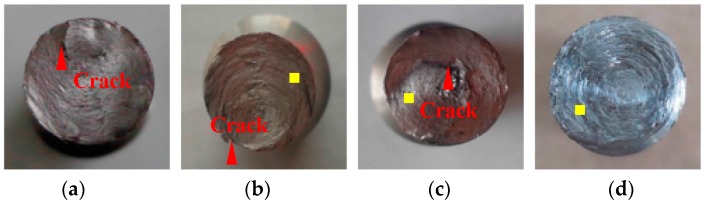
Failure modes under torsion. (**a**) Q235; (**b**) AL6061; (**c**) SS316; (**d**) LYS160.

No necking was observed in the monotonic torsion tests (Figure 6). The fracture sections were almost perpendicular to the axial direction. Cracks or steps could be found in the middle and edges of the fracture sections of the Q235, AL6061, and SS316 specimens (Figure 6a–c). The fracture surface of LYS160 specimen (Figure 6d) was very smooth, while the fracture surfaces of the other specimens were very rough. Instead of cracks or steps, many filamentous concentric circles were distributed homogenously on the fracture surface of LYS160. In addition, the cracks between the circles were rarely small.

**Figure 7:**
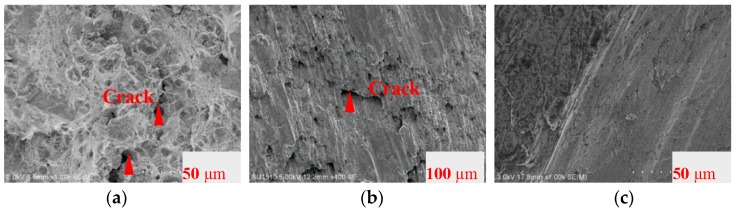
SEM images of the fracture sections under shear. (**a**) AL6061; (**b**) SS316; (**c**) LYS160.

The fracture surfaces of AL6061, SS316, and LYS160 were further observed by SEM (Figure 7). The flat regions within the same approximate diameter (marked with a square in Figure 6b–d) were selected for SEM analysis. The highlighted parts in Figure 7a,b show that brittle fracture was the main failure mode. Moreover, several brittle cracks and a number of cavities were also visible in AL6061 and SS316. Meanwhile, in LYS160, the smooth friction trace between the layers was very clear. The fracture surface of the LYS160 specimen was a slip plane composed of thousands of parallel slip lines.

#### 3.1.2. Mechanical Properties

**Figure 8:**
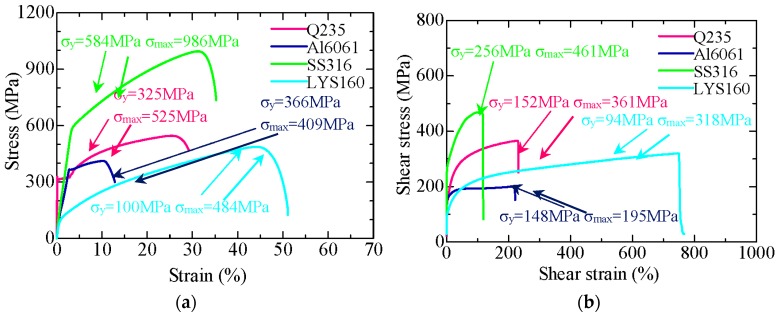
Stress-strain curves under monotonic test. (**a**) Tension test results; (**b**) Torsion test results.

The monotonic stress-strain responses of the materials are displayed in Figure 8. Regardless of the type of test (tension or torsion), the stress-strain curves of the specimens were primarily composed of three parts: elastic range, plastic range, and failure range. No large stiffness differences were observed between the four materials in the tension and the torsion tests. The reduction in the cross-sectional area due to necking caused increased stresses (without increased force) in the plastic range, and the stresses dropped slowly in the failure range (Figure 8a). In contrast, the shear areas were unchanged, and the shear stresses increased with an increase in the torque. The increasing torque leads to instantaneous fracture of the specimens, which is expressed as the sharp drop in the stresses in the failure range (Figure 8b). The tensile yield strength of LYS160 was 225 MPa, which was smaller than that of Q235, while the maximum strength difference between these two materials was narrowed to 41 MPa. A similar trend was observed in the torsional tests.

In the case of tension, although the deformation capacity of SS316 was not the largest, it had merits of high strength (986 MPa) along with large deformation capacity (35%). However, the deformation capacity of SS316 was the lowest in the case of shear. It may be a good choice to design the damper by utilizing the tension and compression of SS316. LYS160 showed the best deformation capacity irrespective of the loading condition. In the case of tension, the ultimate deformation capacity of LYS160 was around 50%, which was almost two, 1.5, and four times that of Q235, SS316, and AL6061, respectively. In the case of torsion, the ultimate deformation capacity of LYS160 was 3.5, five, and four times that of Q235, SS316, and AL6061, respectively. 

**Figure 9:**
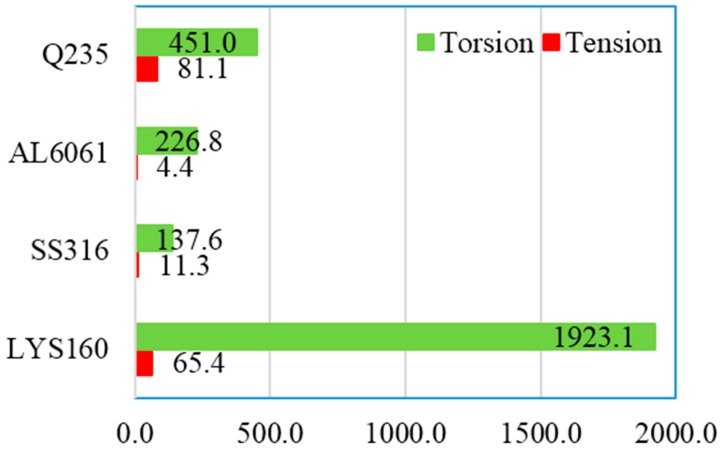
Plastic deformation ratios of the test specimens.

The continuous plastic deformation capacity of the material after the yielding process is very important for the damping material. The plastic deformation ratio *η* (*η* = (*ε*_max_/*ε*_y_) was applied to evaluate the energy dissipation capacity of the material. As shown in Figure 9, the maximum deformation ratio under tension was around 81. Suppose the fatigue cycle is 30, the usable maximum plastic strain is not more than 3*ε*_y_. In the case of torsion, the plastic deformation ratios increased by at least one order of magnitude. For LYS160, the plastic deformation ratio difference was increased by two orders of magnitude. This suggests that the shear loading is more beneficial for utilizing the full deformation capacity and energy dissipation capacity of the material. In the previous studies, the material properties under shear were generally converted from the tensile test results. However, the ultra large plastic deformation capacity (760%) of LYS160 would be covered up by this traditional method of conversion.

### 3.2. Cyclical Torsion Test

#### 3.2.1. Failure Mode

**Figure 10:**
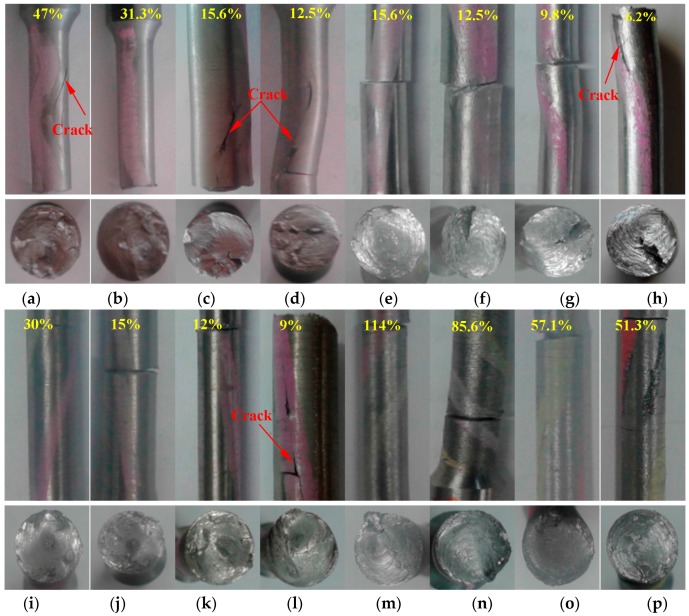
Failure modes under cyclical shear loading. (**a**) Q235-2; (**b**) Q235-3; (**c**) Q235-4; (**d**) Q235-5; (**e**) AL-1; (**f**) AL-2; (**g**) AL-3; (**h**) AL-4; (**i**) SS-1; (**j**) SS-2; (**k**) SS-3; (**l**) SS-4; (**m**) LYS-2; (**n**) LYS-3; (**o**) LYS-4; (**p**) LYS-5.

The failure modes under cyclical torsion tests are presented in Figure 10. The upper part of the figure represents the axial failure mode, while the lower part of the figure represents the fracture surface. During unidirectional torsion, the test specimens were always kept straight. The buckling phenomenon appeared in the Q235 and AL6061 specimens under cyclical loading (Figure 10d,h). This shows that the small crack or the plastic residual deformation in these two materials were likely to trigger the instability under the low cycle fatigue loading. The fracture surfaces of the specimens looked quite different. No large peaks or valleys were observed in the monotonic tests, while they were evident in all the specimens (Figure 10a–l) under cyclical loading, except for LYS160 (Figure 10m–p). Moreover, a large crack was found on the fracture surface of the AL6061 specimen (Figure 10h). This suggests that the failure mode was affected by the fatigue crack, which was mainly caused by the alternating stresses. 

The fracture cross-sections of all the specimens were still perpendicular to the axis. LYS160 was the only specimen in which the fracture surfaces were kept smooth without any cracks. Instead of the filamentous concentric circles found in the monotonic loading tests, several small planes without metallic luster were observed in the cyclical loading tests. The surface exhibited the appearance of a typical ductile failure, resulting from the cyclical accumulated damage caused by interlaminate deformation. One noticeable difference was also discovered between the LYS160 specimens and the other specimens under the cyclical tests. LYS160 only cracked along the radial direction (Figure 10m–p), while all of the conventional materials exhibited cracked along the axial direction.

**Figure 11:**
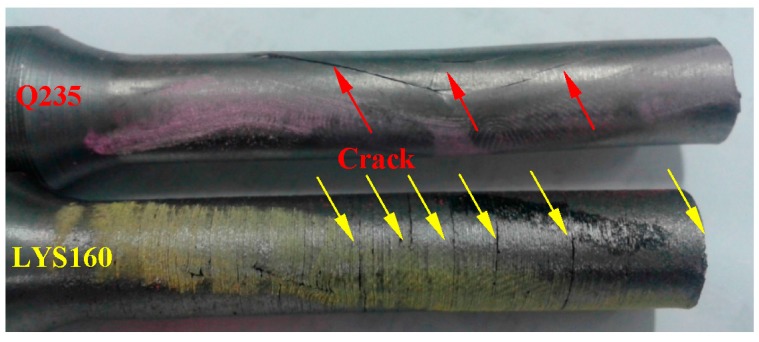
Comparison of the failure mechanism in Q235 and LYS160.

To compare the failure mechanism, enlarged views of the cracks in LYS160 and Q235 are shown in Figure 11. There were two large cracks parallel to the marked line in the Q235 specimen. This suggests that the cracks belong to the opening-mode fracture (I type), which is caused by the cyclical normal stress. Combined with the sliding-mode fracture (II type) at the fracture surface, Q235 exhibited a hybrid fracture mode. The fracture modes of AL6061 and SS316 were similar to that of Q235. As shown in Figure 11, several parallel radial cracks were observed in LYS160. There were also many micro cracks distributed among these visible cracks. The exclusive radial crack direction in LYS160 suggested that shear dominated the failure behavior. From the point of crack distribution, the whole specimen was in the state of interlaminate slip. The crack number and size improved the uniformity of the shear deformation. In view of the above points, the stable uniform interlaminate slip is likely to be produced in LYS160 even under the cyclical loading. Taking advantage of this characteristic, high deformation capacity and energy dissipation capacity could be obtained.

#### 3.2.2. Hysteretic Curve

**Figure 12:**
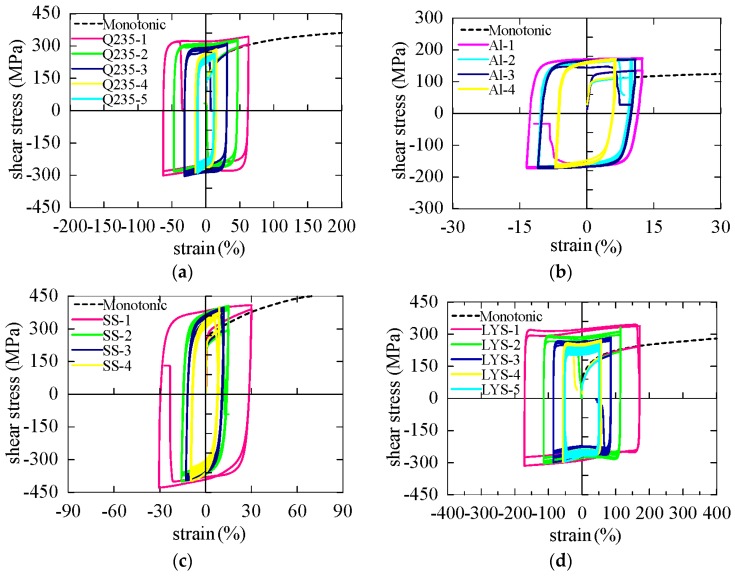
Hysteretic curves of the test specimens. (**a**) Q235; (**b**) AL6061; (**c**) SS316; (**d**) LYS160.

The hysteresis curves of the specimens are shown in Figure 12. The experimental results clearly showed that all the materials exhibit a good hysteretic curve, with a stable and saturated shape. The hysteretic curves of Q235, AL6061, and SS316 were spindle shaped. When the plastic strain amplitude was small, the overall shape of the plastic region was like an arc. With the increase of the strain amplitude, the arcs were shortened gradually. When the strain amplitudes of Q235 and SS316 were 62.8% and 30%, respectively, the transition arcs between the elastic range and the plastic range were negligible and the shape of the hysteresis became like a parallelogram. In contrast, regardless of the strain amplitude, the shape of the hysteretic curve for LYS160 was rectangular.

The elastic stiffness values of all four materials were approximately 200 MPa. After the elastic process, ductile behavior was observed where the post-yield stiffness reduced sharply. The post-yield stiffness (<5 MPa) was much smaller than the elastic stiffness. The post-yield stiffness of LYS160 was always close to 0 in this study. Similar to LYS160, AL6061 exhibited almost perfect elastic–plastic deformation. Hence, the bilinear model with zero post-yield stiffness is suitable for describing the shear behavior of these two materials. When the response strain amplitudes of Q235 and SS316 were larger than 30%, the bilinear model was applicable by appropriate adjustment of the post-yield stiffness.

The monotonic torsion test results (see the dash lines) were also inserted in the respective hysteretic curves for comparing the mechanical property. The yield strength under cyclical loading was generally larger than that under monotonic loading. The Bauschinger effect was also more or less observed in all the tests. The reversal yield strength was smaller than the former yield strength in the opposite loading direction. Since the strain amplitude was the same, the magnitudes of the maximum and minimum stresses were almost the same, and no obvious cyclical softening or hardening was observed. Kinematic hardening was evident in the hysteretic curves of Q235 and SS316. Besides the kinematic hardening, small isotropic hardening also existed in Q235. On the contrary, both kinematic hardening and isotropic hardening were negligible in the hysteretic curves of AL6061 and LYS160. As the loading strain amplitudes and the amplitude differences were both relatively small, the isotropic hardening was not easy to be observed in AL6061. Since the loading strain amplitudes and the amplitude differences for LYS160 were 10 times larger than those of AL6061, the isotropic hardening demonstrated in the figure was also relatively small.

#### 3.2.3 Low Cycle Fatigue

##### Strain-Life Fatigue Curve

**Figure 13:**
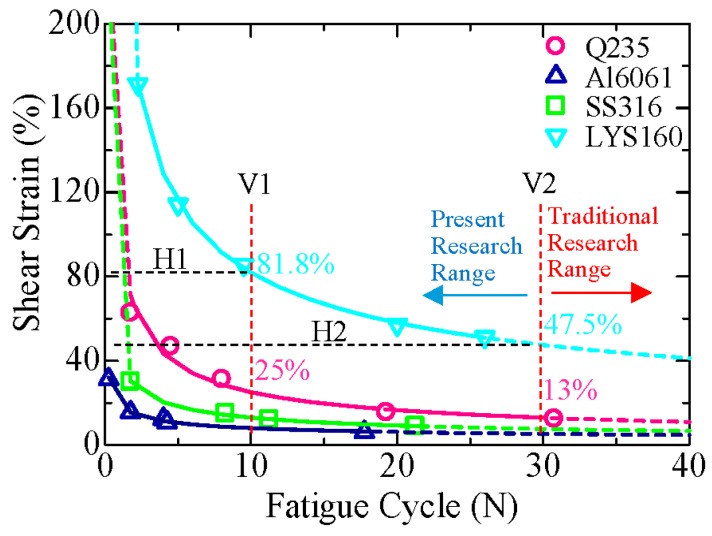
Fatigue curves of the test specimens.

The phenomenon of sudden fracture is the material failure mode under torsion loading, just as necking is considered as the failure mode under tension loading. Therefore, the number of fatigue cycles to fracture was set when the stress begins to drop. The deformation capacity is the most important parameter for the lightweight design of the damper. Hence, instead of stress, the strain-fatigue curve was plotted as shown in Figure 13. The abscissa represented the fatigue cycles and the ordinate represented the shear strain amplitude. Similar to the traditional fatigue curve, the strain-fatigue curve was composed of three parts: steep drop area, transition area and flat area. In the steep drop area (left dash lines of the fitting curves), the strain amplitude dropped dramatically while the fatigue cycle was small in number. On the contrary, even a small drop in the strain amplitude leads to a sharp increase in the number of fatigue cycles in the flat area (right dash lines of the fitting curves). The fit curves of the test results, which are shown as solid lines in the figure, were located in the transition area. The Miner rule was not suitable for evaluating the material fatigue performance in this area. The strain-life fatigue curves exhibited the nonlinear property, which can be expressed as follows:

LYS160: *γ* = 255.88(N_f_)^−0.495^ (3)

Q235: *γ* = 99.577(N_f_)^−0.598^ (4)

Al6061: *γ* = 39.874(N_f_)^−0.485^ (5)

SS316: *γ* = 19.147(N_f_)^−0.375^ (6)

##### Applicable Strain Amplitude

The traditional studies have focused on the fatigue life cycles greater than 30. However, the cyclical shear behavior of the metallic damping material under large plastic response was studied here for the first time. The fatigue cycle ranging from 10 to 30 was selected from the viewpoint of practical applications. It is evident from Figure 13 that the fatigue performances of SS316 and AL6061 are very poor. The fatigue performance of Q235 was better than the aforementioned two materials, but it was still far poorer than that of LYS160, which exhibited an overwhelmingly superior fatigue performance. 

When the fatigue cycle was 10 (Dash line V1), the applicable strain amplitude of LYS160 was 81.8%, which was around 3.27 times that of Q235 (25%). The corresponding applicable strain amplitudes of SS316 and AL6061 were only 13% and 8%, respectively. The applicable strain amplitude of LYS160 was 10 times that of AL6061. When the fatigue cycle was 30 (Dash line V2), the applicable strain amplitudes of SS316 and AL6061 were far smaller than the value for Q235 (13%). The applicable strain amplitude of LYS160 dropped from 81.8% to 47.5%, which was still 3.65 times that of Q235. In addition, the ultimate strain amplitude of LYS160 was around 3.3 times that of Q235 in the monotonic shear test. Supposing the fatigue cycles were the same, the multiple differences in the amplitude had little change in the present research range. This indicates that, under the condition that the fatigue cycles were not more than 30, the amplitude difference between Q235 and LYS160 under cyclical loading can be evaluated from the amplitude difference under monotonic loading, regardless of the effect of the fatigue cycle.

##### Fatigue Cycles

Adequate low cycle fatigue performance of the metallic damping material is very important. Hence, the fatigue performances of the two most popular materials, LYS160 and Q235, were compared with each other. When the strain amplitude was 81.8% (Dash line H1), the corresponding number of fatigue cycles of LYS160 and Q235 were 10 and two, respectively. The difference between these two materials was a factor of 5. This increased to a factor of 7.5 when the strain amplitude was 47.5% (Dash line H2), with the respective fatigue cycles being 30 and four for LYS160 and Q235, respectively. With the decrease of the strain amplitude, LYS160 had an increased fatigue performance advantage over Q235. Taking the consideration of the application, the fatigue performance of LYS160 was at least five times higher than that of Q235.

In addition, the ultimate shear strain of LYS160 was considered as the elongation under monotonic tension, which was around 50%. Meanwhile, when 50% shear strain was adopted in the cyclical shear loading test, the corresponding fatigue cycle was 26. This suggests that the applicable strain amplitude or the fatigue performance of LYS160 is seriously underestimated by the simple conversion from the coupon test results.

##### Accumulated Energy

**Figure 14:**
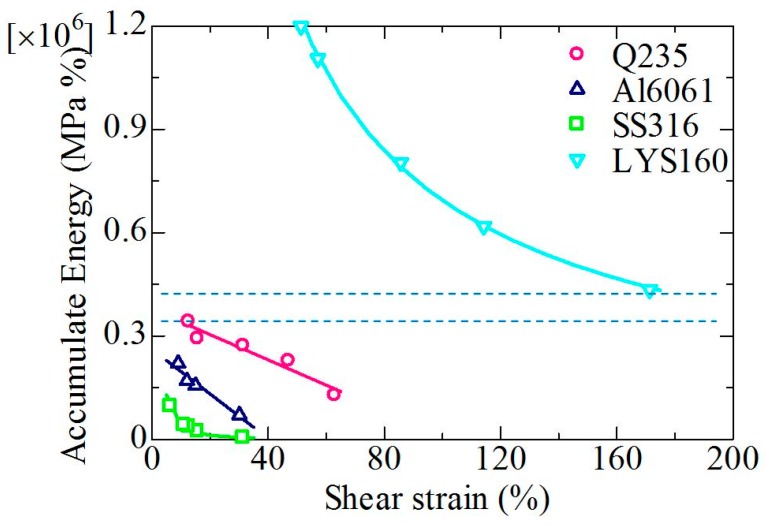
Accumulated energy.

Accumulated energy (AE) is also an important factor to evaluate the seismic performance of the damper. Normalized stress-strain energy (AE = stress × strain, MPa%) was adopted to evaluate the damper performance. The horizontal axis represents the strain amplitude, while the vertical axis represents the accumulated energy. As shown in Figure 14, the accumulated energies of the three traditional materials were relatively concentrated, and the value for Q235 was the largest among the three materials. A large gap was observed between LYS160 and the conventional materials. When the strain amplitude of LYS160 was 171.3%, the AE was 4.3 × 10^5^ (MPa%), which was still larger than the maximum AE of Q235 (two dashed lines). When the strain amplitude was 51.3%, the AE of LYS160 was 1.2 × 10^6^ (MPa%), which was five times that of Q235. The AE of Q235 and AL6061 decreased linearly with the increase of the strain amplitude. The accumulated energy-strain curves of LYS160 and SS316 were nonlinear. Hence, the Miner rule was still unsuitable for the AE evaluation. The accumulated energy-strain relation can be expressed as follows:

LYS160: AE = 3 × 10^7^*γ*^−0.842^ (7)

Q235: AE = −3665.9*γ* + 377,502 (8)

Al6061: AE = −6475.4*γ* + 261,642 (9)

SS316: AE = 2 × 10^6^*γ*^−1.703^ (10)

### 3.3. Stability and Failure Mechanism

The test results proved the instability of the traditional materials under cyclical shear. However, LYS160 always maintained perfect stability, irrespective of the loading condition (monotonic shear or cyclical shear) with large plastic strain. There was a significant difference between the failure mechanism of LYS160 and those of the traditional materials.

The shear deformation of LYS160 can be regarded as slippage among numerous parallel circular planes. The good stability of the slippage was attributed to the simple shear loading, the two fixed ends of the specimens, zero axial deformation, and the normal vector of the restrained slip plane. In other words, shear deformation is likely to be produced under simple shear loading. As the loading condition of the SPD is similar to the torsional tests, the shear deformation should be incorporated into the buckling shape evaluation, and the traditional buckling analysis method for predicting the stability should be reconsidered for LYSPD.

## 4. Conclusions

Axial and torsional specimens of four types of metallic damping materials were tested in this study. The material properties including the mechanical performance, plastic response and the failure mechanism under large repeated plastic shear loading as well as low cycle fatigue were investigated in detail. This study provided a reference for the material properties which are important for lightweight design. The main results are summarized as follows:

(1) The deformation capacity of LYS160 was underestimated by the conversion from the traditional tensile test. The maximum shear strain of LYS160 is 760%, which is more than 25 times that of the elongation.
(2) When the fatigue cycles are 10 and 30, the applicable shear strain amplitudes of LYS160 are 81.8% and 47.5%, respectively. The fatigue life of LYS160 is at least five times that of Q235 when the same strain amplitude is adopted in the cyclical shear loading.
(3) Owing to the small transition between the elastic and plastic region, the perfect elastic-plastic model can describe the mechanical property of LYS160 well, under large repeated plastic shear loading. The nonlinear hardening should be considered in the case of Q235 as its applicable shear strain amplitude is not more than 25%.
(4) Dimple and slip plane are the failure behaviors of LYS160 under the tension and the shear loading, respectively, which are characterized as the plastic fracture. It is totally different from the failure mechanism of the conventional metallic materials.

## Figures and Tables

**Table 1:** Details of the monotonic test specimens.

No.	Name	Diameter (mm)	Length (mm)	Test Type	
				Tension	Torsion
1	Q235	10	100	Ten1	Tor1
2	Q235			Ten2	Tor2
3	Q235			Ten3	Tor3
4	AL6061			Ten4	Tor4
5	AL6061			Ten5	Tor5
6	AL6061			Ten6	Tor6
7	SS316			Ten7	Tor7
8	SS316			Ten8	Tor8
9	SS316			Ten9	Tor9
10	LYS160			Ten10	Tor10
11	LYS160			Ten11	Tor11
12	LYS160			Ten12	Tor12

**Table 2:** Details of the cyclical shear test specimens.

No.	Material	Name	Shear Strain	Fatigue Cycle
			(%)	(Nf)
1	Q235	Q235-1	62.8	1.75
2		Q235-2	47.0	4.50
3		Q235-3	31.3	8.00
4		Q235-4	15.6	19.25
5		Q235-5	12.5	30.75
6	AL6061	AL-1	15.6	1.75
7		AL-2	12.5	4.00
8		AL-3	9.8	4.25
9		AL-4	6.2	17.75
10	SS316	SS-1	30.0	1.75
11		SS-2	15.0	8.25
12		SS-3	12.0	11.25
13		SS-4	9.0	21.25
14	LYS160	LYS-1	171.4	2.25
15		LYS-2	114.2	5.00
16		LYS-3	85.6	9.50
17		LYS-4	57.1	20.00
18		LYS-5	51.3	26.00

## References

[1] Takeuchi T., Hajjar J.F., Matsui R., Nishimoto K., Aiken I.D. (2012). Effect of local buckling core plate restraint in buckling restrained braces. Eng. Struct..

[2] Vasdravellis G., Karavasilis T.L., Uy B. (2013). Large-scale experimental validation of steel posttensioned connections with web hourglass pins. J. Struct. Eng..

[3] De Matteis G., Landolfo R., Mazzolani F.M. (2003). Seismic response of MR steel frames with low-yield steel shear panels. Eng. Struct..

[4] Mistakidis E.S., de Matteis G., Formisano A. (2007). Low yield metal shear panels as an alternative for the seismic upgrading of concrete structures. Adv. Eng. Softw..

[5] Estrada I., Real E., Mirambell E. (2008). A new developed expression to determine more realistically the shear buckling stress in steel plate structures. J. Constr. Steel Res..

[6] Brando G., de Matteis G. (2011). Experimental and numerical analysis of a multi-stiffened pure aluminium shear panel. Thin Walled Struct..

[7] Deng K., Pan P., Li W., Xue Y. (2015). Development of a buckling restrained shear panel damper. J. Constr. Steel Res..

[8] Brando G., D’Agostino F., de Matteis G. (2013). Experimental tests of a new hysteretic damper made of buckling inhibited shear panels. Mater. Struct..

[9] Deng K., Pan P., Su Y., Ran T., Xue Y. (2014). Development of an energy dissipation restrainer for bridges using a steel shear panel. J. Constr. Steel Res..

[10] Rai D.C., Annam P.K., Pradhan T. (2013). Seismic testing of steel braced frames with aluminum shear yielding dampers. Eng. Struct..

[11] Brando G., de Matteis G. (2014). Design of low strength-high hardening metal multi-stiffened shear plates. Eng. Struct..

[12] Chen Z., Dai Z., Huang Y., Bian G. (2013). Numerical simulation of large deformation in shear panel dampers using smoothed particle hydrodynamics. Eng. Struct..

[13] Piedrafita D., Cahis X., Simon E., Comas J. (2013). A new modular buckling restrained brace for seismic resistant buildings. Eng. Struct..

[14] Abebe D.Y., Jeong S.J., Getahune B.M., Segu D.Z., Choi J.H. (2015). Hysteretic characteristics of shear panel damper made of low yield point steel. Mater. Res. Innov..

[15] Bouvier S., Haddadi H., Levée P., Teodosiu C. (2006). Simple shear tests: Experimental techniques and characterization of the plastic anisotropy of rolled sheets at large strains. J. Mater. Process. Technol..

[16] Zhang C., Zhang Z., Shi J. (2012). Development of high deformation capacity low yield strength steel shear panel damper. J. Constr. Steel Res..

[17] Liu Y., Shimoda M. (2013). Shape optimization of shear panel damper for improving the deformation ability under cyclic loading. Struct. Multidisc. Optim..

[18] Zhang C., Zhang Z., Zhang Q. (2012). Static and dynamic cyclic performance of a low-yield-strength steel shear panel damper. J. Constr. Steel Res..

[19] Zhang C., Aoki T., Zhang Q., Wu M. (2013). Experimental investigation on the low-yield-strength steel shear panel damper under different loading. J. Constr. Steel Res..

[20] Dusicka P., Itani A.M., Buckle I.G. (2007). Cyclic response of plate steels under large inelastic strains. J. Constr. Steel Res..

[21] Tateishi K., Hanji T., Minami K. (2007). A prediction model for extremely low cycle fatigue strength of structural steel. Int. J. Fatigue.

[22] De Matteis G., Mazzolani F.M., Panico S. (2008). Experimental tests on pure aluminium shear panels with welded stiffeners. Eng. Struct..

[23] Nakashima M., Iwai S., Iwata M., Takeuchi T., Konomi S., Akazawa T., Saburi K. (1994). Energy dissipation behavior of shear panels made of low yield steel. Earthq. Eng. Struct. Dyn..

[24] Nip K.H., Gardner L., Davies C.M., Elghazouli A.Y. (2010). Extremely low cycle fatigue tests on structural carbon steel and stain-less steel. J. Constr. Steel Res..

[25] Zhang C., Zhu J., Wu M., Yu J., Zhao J. (2016). The lightweight design of a seismic low-yield-strength steel shear panel damper. Materials.

